# 300. Long COVID in Cancer Patients: Preponderance of Symptoms in Majority of Patients Over Long Time Period

**DOI:** 10.1093/ofid/ofab466.502

**Published:** 2021-12-04

**Authors:** Hiba Dagher, Alexandre Malek, Anne-Marie Chaftari, Ishwaria M Subbiah, Ying Jiang, Peter Lamie, Bruno Granwehr, Teny John, Eduardo Yepez Guevara, Jovan Borjan, Cielito Reyes, Mary Flores, Fareed Khawaja, Mala Pande, Norman Ali, Raniv Rojo, Daniel D Karp, Ray Y Hachem, Issam I Raad

**Affiliations:** 1 UT MD Anderson Cancer Center, Houston, Texas; 2 The University of Texas MD Anderson Cancer Center, Houston, Texas; 3 MD Anderson Cancer Center, Houston, TX; 4 The University of Texas MD Anderson Cancer Center, Houston, Texas, Houston, TX; 5 University of Texas MD Anderson Cancer Center, Houston, Texas

## Abstract

**Background:**

An increasing number of observational studies have reported the persistence of symptoms following recovery from acute COVID-19 disease. The long-term consequences of COVID-19 are not fully understood and there is no clear consensus on the definition of post-acute sequelae of SARS-CoV-2 infection (PASC). The reported prevalence of PASC widely varies from 10% up to 87%. The purpose of this study is to assess PASC in cancer patients following acute COVID-19 recovery.

**Methods:**

We assessed cancer patients at MD Anderson Cancer Center who were diagnosed with COVID-19 disease between March 1, 2020 and Sept 1, 2020. Using patient questionnaires and medical chart reviews we followed these patients from March 2020 till May 2021. Patient questionnaires were sent out remotely daily for 14 days after COVID-19 diagnosis then weekly for 3 months, and then monthly thereafter. Chart reviews were conducted for each patient hospital re-admission and emergency department visit. These admissions were classified as either COVID-19 related or non-related. The persistence or emergence of new COVID19-related symptoms were captured at each COVID-19 related admission.

**Results:**

We included 312 cancer patients with a median age of 57 years (18-86). The majority of patients had solid tumors (75%). Of the 312 patients, 188 (60%) reported long COVID-19 symptoms with a median duration of 7 months and up to 14 months after COVID-19 diagnosis. The most common symptoms reported included fatigue (82%), sleep disturbances (78%), myalgias (67%) and gastrointestinal symptoms (61%), followed by headache, altered smell or taste, dyspnea (47%) and cough (46%). A higher number of females reported a persistence of symptoms compared to males (63% vs 37%; p=0.036). Cancer type, neutropenia, lymphocytopenia, and hospital admission during acute COVID-19 disease were comparable in both groups and did not seem to contribute to a higher number of long-COVID-19 patients in our study group.

**Conclusion:**

Long-COVID occurs in 60% of cancer patients and may persist up to 14 months after acute illness. The most common symptoms are fatigue, sleep disturbance, myalgia and gastro-intestinal symptoms.

**Disclosures:**

**Fareed Khawaja, MBBS**, **Eurofins Viracor** (Research Grant or Support)

